# Social media as a tool for assessing patient perspectives on quality of life in metastatic melanoma: a feasibility study

**DOI:** 10.1186/s12955-018-1047-z

**Published:** 2018-11-29

**Authors:** Amr Makady, Rachel R. J. Kalf, Bettina Ryll, Gilliosa Spurrier, Anthonius de Boer, Hans Hillege, Olaf H. Klungel, Wim Goettsch, on behalf of GetReal Workpackage 1

**Affiliations:** 1 Zorginstituut Nederland, Eekholt 4, 1112 XH Diemen, The Netherlands; 2 Department of Pharmacoepidemiology and Clinical Pharmacology, Universiteit Utrecht, Utrecht, The Netherlands; 3 Melanoma Patient Network Europe, Uppsala, Sweden; 4 Uppsala University, Uppsala, Sweden; 5 Department of Epidemiology, University Medical Centre Groningen, Groningen, The Netherlands

**Keywords:** Social media, Health related quality of life, Real-world data, Patient perspectives

## Abstract

**Purpose:**

Development of innovative drugs for melanoma is occurring rapidly. Incremental gains in overall survival amongst innovative products may be difficult to measure in clinical trials, and their use may be associated with increased toxicity profiles. Therefore, HTA agencies increasingly require information on HRQoL for the assessment of such drugs. This study explored the feasibility of social media to assess patient perspectives on HRQoL in melanoma, and whether current cancer- and melanoma-specific HRQoL questionnaires represent these perspectives.

**Methods:**

A survey was distributed on the social media channels of Melanoma Patient Network Europe to assess melanoma patients’ perspectives regarding HRQoL. Two researchers independently conducted content analysis to identify key themes, which were subsequently compared to questions from one current cancer-specific and two melanoma-specific HRQoL questionnaires (i.e. EORTC QLQ-C30, EORTC QLQ-MEL38, FACT-M).

**Results:**

In total, 72 patients and 17 carers completed the survey. Patients indicated that family, having a normal life, and enjoying life were the three most important aspects of HRQoL for them. Carers indicated that being capable, having manageable adverse events, and being pain-free were the three most important aspects of HRQoL for patients. Respondents seem to find some questions from HRQoL questionnaires relevant (e.g. ‘Have you felt able to carry on with things as normal?’) and others less relevant (e.g. ‘Have you had swelling near your melanoma site?’). Additionally, wording may differ between patients and HRQoL questionnaires, whereby patients generally use a more positive tone.

**Conclusions:**

Social media may provide a valuable tool in assessing patient perspectives regarding HRQoL. However, differences seem to emerge between patient and carer perspectives. Additionally, patient perspectives did not seem to fully correlate to questions posed in cancer- (i.e. EORTC QLQ-C30) and melanoma-specific (i.e. EORTC QLQ-MEL38, FACT-M) HRQoL questionnaires examined.

**Electronic supplementary material:**

The online version of this article (10.1186/s12955-018-1047-z) contains supplementary material, which is available to authorized users.

## Introduction

Health Technology Assessment (HTA) entails the systematic evaluation of the properties and effects of health technologies, addressing their direct and intended effects, as well as their indirect and unintended consequences with the aim of informing decision-making [1]. In multiple jurisdictions, manufacturers of (new) health technologies need to provide evidence that their product is of equal- or additional benefit to those currently available in order to qualify for reimbursement. Public or private HTA agencies then conduct an HTA of a submission (i.e. dossier of evidence) provided by the manufacturer to assess the (additional) benefit of the health technology. Subsequently, national, regional or local decision-makers (such as Ministries of Health, national payers or local payers) will use this HTA for their decision on reimbursement. Such HTA assessments can encompass several aspects of the implementation of health technologies in clinical practice such as their relative effectiveness, cost-effectiveness or appropriate use [1]. Relative effectiveness is defined as the extent to which an intervention does more good than harm, when compared to one or more alternative interventions for achieving the desired results and when provided under the routine setting of health care practice [2].

Within the field of oncology, the development of innovative yet expensive therapeutic drugs is occurring at a rapid pace. Metastatic Melanoma provides an example where 8 novel therapies and 3 combination therapies, which belong to three new therapeutic classes, have gained market authorisation since 2011. [3, 4]. One positive consequence of the increased number of treatments has been the general prolongation of overall survival of metastatic melanoma patients [3, 4]. However, provided that incremental gains in overall survival associated with innovative products may be difficult to measure in the context of clinical trials, and the toxicity profiles associated with their use may be considerable, HTA agencies increasingly require information on health-related quality of life (HRQoL) experienced by patients during the prolonged periods of survival as a means to assess the added value of innovative products within relative effectiveness assessments (REA) [5–7].

Conventionally, HRQoL of patients is measured using validated questionnaires that can be generic (e.g. EQ-5D) [8], disease-specific (e.g. EORTC-QLQ-30 or FACT-M) [9, 10] or that include additional individualised measures [11]. From an HTA perspective, HRQoL data generated by generic questionnaires offers the advantage of allowing for comparison of health gains across disease areas (e.g. oncology vs. chronic pulmonary diseases). Meanwhile, data acquired through disease-specific questionnaires aims to distinguish between HRQoL experienced at different stages of a particular disease (e.g. metastatic melanoma), thus possibly identifying medical need per disease stage. Therefore, HRQoL data can contribute to HTA as primary or secondary health outcomes for relative effectiveness data or as sources for utility values used in cost-utility analyses of new oncologic treatments [5–7].

However, despite HTA guidance encouraging the collection of HRQoL data for HTA submissions, it is seldom included in submissions. Recent research in 6 different European jurisdictions has shown that HRQoL data features in only a third of HTA submissions for oncological treatments, with limited impact on decision-making for a number of reasons, including its sheer scarcity [5]. In addition, the available validated HRQoL questionnaires, whether generic or disease-specific, generally show low completion rates by patients, despite a generally prevailing notion of the importance of HRQoL. [12–14].

The IMI-GetReal initiative is a 3-year public-private partnership exploring the use of Real-World Evidence (RWE) in early drug development and drug assessment [15], including a series of case studies. Here presented is our case study on metastatic melanoma where the potential of social media as a new source of RWE for HTA was investigated within a pilot literature review [12, 16]. This research demonstrated the potential value of using social media to inform several parameters of HTA in oncology, including: adverse events [12, 17–19], treatment adherence [18] and HRQoL [12, 20–22].

Building upon results from this pilot review, this article aims to explore the use of social media as a tool to gather melanoma patients´ perspectives on HRQoL. More specifically, this article will: assess the comparability of the melanoma patient population accessible via social media with the general melanoma population, evaluate what melanoma patients and carers perceive as important in relation to HRQoL and compare this to validated cancer-specific HRQoL questionnaires, and assess whether current melanoma-specific HRQoL questionnaires represent melanoma patients’ perspectives on HRQoL. It is important to emphasize that this is a feasibility study, aiming to advance the science of using social media to gain insights on patients’ perspectives on HRQoL, rather than conducting a robust quantitative analysis to answer pre-defined hypotheses based on data collected through social media.

## Materials and methods

Members of Melanoma Patient Network Europe (MPNE) [23], an established patient network for melanoma patients, carers and advocates, were approached via multiple social media channels of MPNE to anonymously complete a web-based survey. An announcement with a brief description of the survey goals and link to the survey was posted on the private MPNE Facebook group, MPNE LinkedIn group, and MPNE twitter account. Members of MPNE were also approached by sending a single e-mail to the MPNE mailing list and by posting the announcement and link to the survey on the website of MPNE. Respondents were eligible for inclusion in the study if they self-reported a diagnosis of melanoma on the online survey or reported to be carer of a melanoma patient.

The web-based survey was conducted using Survey Monkey, and once a member clicked on the survey link it was presented on a separate screen. The survey was open for 30 days from January 8th 2016. Two reminders of the ongoing survey were posted on MPNE’s private Facebook group, LinkedIn group, and Twitter account throughout the 30 day period. Respondents gave their informed consent by completing the survey. According to the 1964 declaration of Helsinki and its later amendments as well as with the ethical standards of Dutch law [24], no official approval of an Ethical committee was necessary.

The web-based survey included 25 items (see Additional file 1: Appendix 1). Socio-demographic and clinical characteristics were collected, including gender, country of residence, age, educational level, years since melanoma diagnosis, stage of disease, treatments received and patient-reported HRQoL. Patient and carer perspectives on HRQoL were explored by asking several open questions, including:

- What is HRQoL in melanoma for you?
- Name the 3 things that deteriorate your/the melanoma patient’s HRQoL today?
- Name the single thing that would improve your/the melanoma patient’s HRQoL right now?

To assess the comparability and generalizability of our study population to the general melanoma population, we compared socio-demographic variables to reported values in the literature [25–27]. A comparison of educational level was made by using the study of Eriksson et al. [25], where all Swedish patients diagnosed with an invasive cutaneous malignant melanoma between 1990 and 2007 were included. Patients had a median age of 62 years at diagnosis. The age distribution in our study was compared to data available from the EORTC QLQ-C30 reference values [26], these reference values are based on responses from a total of 223 stage I and II melanoma patients and 585 of stage III and IV melanoma patients. The gender distribution in our study was compared to that reported by Bay et al. [27], where all skin melanoma patients registered in the Danish Cancer Register (1989–2011) were included. Additionally, we compared the overall quality of life reported in the EORTC QLQ-C30 reference values for melanoma patients to the overall quality of life reported by our study population. The question in our survey was similar to the question in the EORTC QLQ-C30, namely ‘On a scale from 1 (poor) to 7 (excellent), please rate your/the patient’s Quality of Life today (see Additional file 1: Appendix 1).

In order to evaluate what patients and carers regard important for HRQoL, two researchers independently performed inductive content analysis on the responses to the open-ended questions posed in the survey [28]. Content analysis allows for the organisation and cataloguing of respondent’s descriptions of key aspects regarding melanoma patient views on HRQoL. Assigned codes and the grouping of similar codes were reviewed by both researchers and any discrepancies in coding were resolved by consensus. We present the results of the content analysis in two ways. First, we constructed a top 10 of aspects that are most often mentioned by patients and carers. This provides an insight of what patients and carers deem most important in melanoma patients HRQoL. Second, we created a word cloud based on the frequency generated codes were cited (either by patients or carers) to illustrate which aspects in HRQoL are most often mentioned by patients and carers. A word cloud visually represents the frequency words are mentioned in the text analysed, the more often a word is mentioned the larger in size it will be in the word cloud [29, 30].

To assess the extent to which current cancer-specific HRQoL questionnaires represent melanoma patients’ perspectives on HRQoL, respondents were asked to rate the relevance of questions from the EORTC QLQ-C30 on a 5-point Likert scale, ranging from “not relevant at all” to “very relevant”. The percentage of responses were then calculated per question; stratified for patients per disease stage and including a separate stratum for carers. The EORTC QLQ-C30 is a 30-item questionnaire that assesses the HRQoL of cancer patients and has been translated and validated in 41 languages. This cancer-specific HRQoL questionnaire can be used in conjunction with specific modules that allow the evaluation of HRQoL in specific patient populations (e.g. melanoma, breast cancer, lung cancer) [31].

To assess the relevance of questions in two melanoma-specific HRQoL questionnaires to respondents, namely EORTC MEL-38 and FACT-M, we performed a qualitative comparison of the key aspects identified during the content analysis and compared these to the questions posed in the melanoma-specific HRQoL questionnaires. This made it possible to assess to what extent our study population considered the questions in melanoma-specific HRQoL questionnaires relevant. The EORTC MEL-38 is a 38-item questionnaire that is being developed in a cross-cultural setting and should be used in conjunction with the EORTC QLQ-C30, but has not been validated yet [32]. The FACT-M is a tool including 24 items encompassing three HRQoL domains (i.e. physical well-being, emotional well-being, and social well-being) [33] and has been validated in a population of Stage I to Stage IV melanoma patients [34].

Subgroup analyses were performed for patients and carers separately and were stratified by disease stage when possible. All data were coded, stored and analyzed using R version 4.00.03.05 [35].

## Results

A total of 96 respondents completed the web-based survey. Of these 70 indicated to be patients, 17 were carers of a melanoma patient, 2 indicated to be both patients and carers, and 7 did not report either and were therefore excluded from the analyses. The 2 respondents indicating to be both patients and carers were included in the analysis as patients only. Patients who responded to the survey represented all stages of melanoma; 25% reported to have stage I, 14% reported to have stage II, 22% reported to have stage III, and 39% reported to have stage IV melanoma. Of the carers who responded to the survey, 6% cared for a patient who had stage I, none cared for a patient with stage II melanoma, 24% cared for a patient who had stage III, and most cared for a patient who had stage IV melanoma (71%). All analyses were stratified by stage for patients, however this was not possible for carers due to the small number of respondents.

Most respondents accessed the survey via Facebook (77%), Twitter was used to a lesser extent (2%). Some respondents accessed the survey via the MPNE website (9%) or the MPNE mailing list (1%). Finally, 11% of respondents indicated to have used other online channels linked to the MPNE, such as the Berlin Support Group, Melanoma Romania Association, and Dutch Melanoma Association Forum. The response rate for Facebook was 11%, MPNE had 695 Facebook members and a total of 74 filled in the survey. We were unable to calculate response rates for the other sources on which the survey was distributed, because of the low number of respondents using other social media channels, or social media channels for which we did not possess the relevant information in order to calculate the response rate.

The socio-demographic characteristics of the study population are shown in Table 1. Respondents were mostly female (70%), between 35 and 64 (82%), were university graduates or higher (64%), and originated from the United Kingdom (50%). The paper of Bay et al. showed that approximately 50% of patients with melanoma in Denmark were female [27]; when stratified it was shown that 74% of patients in our study population were female whereas 53% of carers in our study population were female. The distribution of age in our study population was similar for most stages of melanoma and between carers and patients, except for patients with stage III where half of the respondents indicated to be between 55 and 64. The EORTC reference values also showed that the distribution of age was similar between stage I & II and stage III & IV, and were comparable to the age distribution found in our study population [26]. Compared to the educational level reported in the paper by Eriksson et al., where only 25% of melanoma patients in Sweden had a high education (e.g. a college degree or higher), our study sample was more highly educated with 64% having an university degree or higher [25].

**Table 1:** Socio-demographic characteristics of the study population, for each variable the percentages are calculated per stage

	Patients (*n* = 72)				Carers (*n* = 17)	Reference Patient Population^a^				
	Stage I (*n* = 18)	Stage II (*n* = 10)	Stage III (*n* = 16)	Stage IV (*n* = 28)						
Gender:							Stage I	Stage II	Stage III	Stage IV
Male	17%	30%	25%	32%	47%	Male	43%	52%	56%	58%
Female	83%	70%	75%	68%	53%	Female	57%	48%	44%	42%
Age:							Stage I&II	Stage III&IV		
18–24	–	–	–	4%	6%	< 40	25%	27%		
25–34	6%	10%	13%	4%	12%	40–49	23%	26%		
35–44	22%	20%	13%	29%	24%	50–59	24%	23%		
45–54	28%	30%	25%	36%	29%	60–69	22%	18%		
55–64	33%	30%	50%	18%	24%	70–79	5%	6%		
65–74	6%	10%	–	7%	6%	80+	1%	0%		
> 75	6%	–	–	4%	–		0%	0%		
Highest educational level:							All Patients	Female	Male	
Did not attend school	–	–	–	–	–	Low education^b^	36%	36%	36%	
Finished school after primary school	–	–	–	–	–	Intermediate education	39%	39%	39%	
Graduated from secondary school	11%	20%	25%	7%	12%	High education	25%	25%	25%	
Graduated from college	22%	10%	31%	21%	29%					
Graduated with university degree level	50%	60%	38%	50%	35%					
Higher degree or doctorate	17%	10%	6%	21%	24%					
Country of Residence:										
Belgium	–	–	–	11%	6%					
France	–	–	–	4%	6%					
Germany	17%	10%	13%	–	–					
Ireland	–	–	6%	11%	–					
Italy	–	–	6%	–	–					
Netherlands	–	–	6%	7%	24%					
Norway	6%	20%	13%	7%	–					
Other^c^	17%	–	6%	4%	6%					
Romania	11%	10%	–	7%	18%					
UK	50%	60%	50%	50%	41%					

^a^Three socio-demographic characteristics of our study population are compared to results from 3 scientific publications, namely gender [27], age [26], and educational level [25]; − no respondents ticked this answer (e.g. 0%); ^b^In this reference it is given that in Sweden low education corresponds to mandatory school, intermediate to high school, and high to college/university; ^c^5 respondents originated from the USA and 1 respondent from Serbia

Table 2 shows that more than 60% of the patients with stage II, III, and IV melanoma indicated to have been diagnosed more than 2 years ago, compared to 44% of patients with stage I. A total of 65% of the carers indicated to take care of a patient who had been diagnosed more than 2 years ago. Most patients with stage II, III and IV melanoma as well as the carers who responded to the survey indicated that the patient had been diagnosed with cutaneous melanoma. Cutaneous melanoma had been diagnosed in 44% of patients with stage I melanoma, while 50% had been diagnosed with ocular, uveal or choroidal melanoma, and 6% of these patients didn’t know the type of melanoma they had been diagnosed with. Most patients with stage I, II, or III melanoma indicated to be unfamiliar with any mutations present in their tumour, compared to 46% of patients with stage IV melanoma and 53% of carers who indicated that a BRAF mutation had been found in the tumour. Surgery was the treatment most often received by patients according to 92% of patients and carers in this study.

**Table 2:** Clinical characteristics of the study population, for each variable the percentages are calculated per stage

	Patients (*n* = 72)				Carers (*n* = 17)^c^
	Stage I (*n* = 18)	Stage II (*n* = 10)^a^	Stage III (*n* = 16)^b^	Stage IV (*n* = 28)	
Melanoma diagnosis:					
< 1 month ago	–	–	–	–	–
1–3 months ago	6%	–	–	–	–
3–6 months ago	17%	–	7%	4%	–
6–12 months ago	28%	10%	13%	11%	6%
1–2 years ago	6%	20%	7%	25%	29%
2–5 years ago	22%	50%	40%	43%	24%
> 5 years ago	22%	20%	33%	18%	41%
Type of Melanoma:^d^					
Cutaneous melanoma	44%	70%	57%	64%	62%
Ocular/ Uveal/Choroidal melanoma	50%	10%	7%	18%	12%
Acral melanoma	–	–	–	–	6%
Mucosal melanoma	–	–	–	–	–
I don’t know	6%	20%	36%	18%	19%
Melanoma mutations:					
BRAF mutant	11%	20%	27%	46%	53%
BRAF wild-type	–	–	–	18%	18%
NRAS mutant	–	–	–	7%	–
c-kit mutant	–	–	–	4%	6%
GNAQ/GNA11	–	–	7%	4%	–
I don’t know	78%	60%	67%	11%	18%
None	6%	10%	–	4%	6%
Other^e^	6%	10%	–	7%	–
Treatments received^f^					
Surgery	89%	90%	94%	89%	100%
Radiotherapy	39%	20%	13%	39%	26%
Chemotherapy	–	11%	6%	25%	21%
Immune Therapies	–	–	25%	81%	56%
Targeted Therapies	–	–	6%	27%	50%

^a^The total number of respondents on treatments received (chemotherapy) was 9; ^b^The total number of respondents on melanoma diagnosis is 15, on type of melanoma is 14, melanoma mutation is 15, and on treatments received is 15; ^c^Carers provided disease specific characteristics for the patient they care(d) for; ^d^The total number of respondents on type of melanoma is 16, on treatments received is 16; − no respondents ticked this answer (e.g. 0%); ^e^Other melanoma mutations mentioned by 4 respondents were mutations in chromosome 3, 6 and/or 8; ^f^the percentage for ‘treatments received’ could be more than 100% because patients may have received more than one treatment; NA: Not Applicable

The overall HRQoL values reported by our study population are comparable to that reported by the EORTC as reference values for melanoma patients for the EORTC QLQ-C30 (Table 3) [26]. Both stratified and non-stratified overall HRQoL values were similar, indicating that the HRQoL in our study population is similar to that in the general melanoma population.

**Table 3:** Overall quality of life in the study population compared to the EORTC reference value for the EORTC QLQ-C30

	Overall quality of life						
	1	2	3	4	5	6	7
Respondents: Stage I & II (*n* = 28)	0%	0%	7%	11%	21%	36%	25%
EORTC Reference Value: Stage I & II	0%	1%	6%	16%	28%	31%	20%
Respondents: Stage III & IV (*n* = 44)	2%	2%	11%	11%	27%	25%	20%
EORTC Reference Value: Stage III & IV	2%	2%	6%	18%	30%	26%	18%
Respondents: All patients (*n* = 72)	1%	1%	10%	11%	25%	29%	22%
EORTC Reference Value: All Stages	2%	2%	8%	17%	28%	27%	16%

**Fig. 1:**
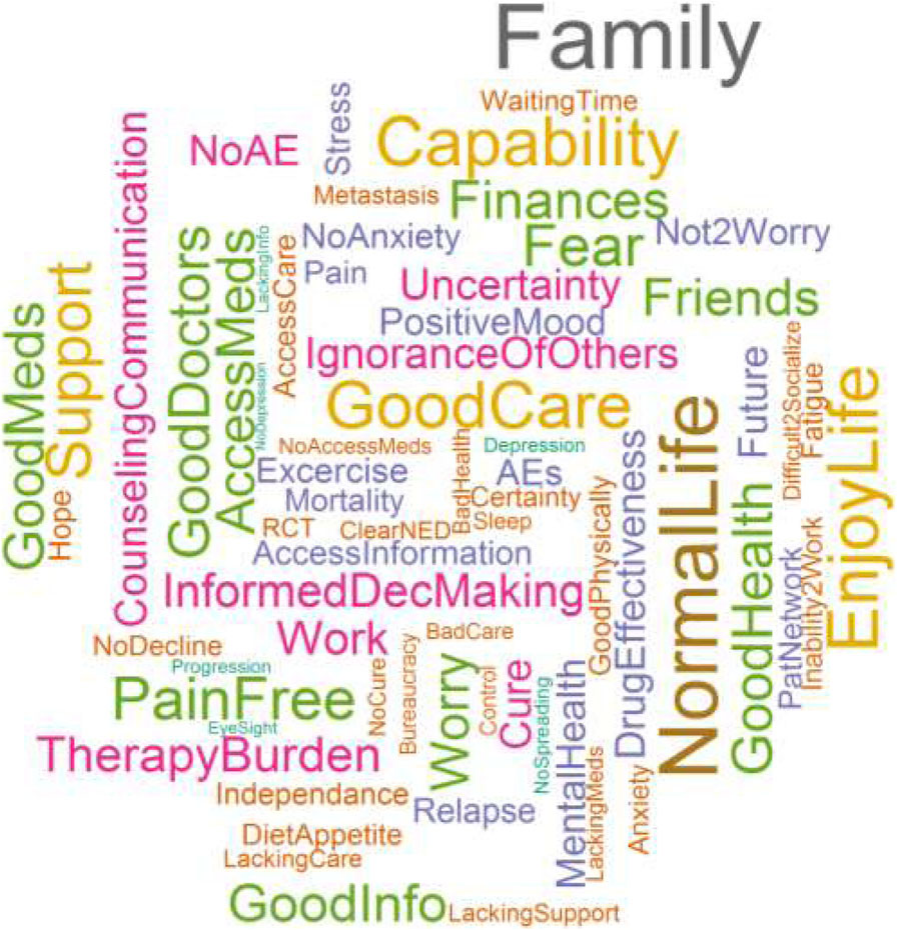
Key aspects patients find important in QoL

Figure 1 shows the most often mentioned aspects by patients and carers in our study sample that are of influence to the patients’ HRQoL. ‘Family’ was the single most often mentioned aspect while the second most often mentioned aspect relevant to melanoma patients’ HRQoL was ‘Normal Life’, implying that patients find it highly important to lead a normal life while being ill. In Table 4 a more detailed analysis is shown whereby the top 10 most often mentioned aspects that influence melanoma patients’ HRQoL is presented. It can be seen that patients themselves most often mentioned ‘Family’ as most important in their HRQoL, together with ‘Good Care’ by patients with stage I melanoma, ‘Fear’ by patients with stage II melanoma, ‘Worry’ by melanoma patients with stage III, and ‘Good medicines’ and ‘Normal Life’ by patients with stage IV melanoma. Carers mentioned ‘Capability’, ‘No Adverse Events’, and ‘Pain free’ most often as important aspects to patients’ HRQoL. The second most often mentioned aspect by carers was ‘Family’, which indicates that patients and carers may have a different perspective regarding what is of most influence in patients’ HRQoL.

**Table 4:** Top 10 aspects mentioned most often by patients and carers as important in patients’ HRQoL

Patients (*n* = 72)				Carers (*n* = 17)
Stage I (*n* = 18)	Stage II (*n* = 10)	Stage III (*n* = 16)	Stage IV (*n* = 28)	
Family^a^	Family^a^	Family^a^	Family^a^	Capability^a^
Good care^a^	Fear^a^	Worry^a^	Good medicines^a^	No AEs^a^
Finances^b^	Enjoy life	Normal life	Normal life^a^	Pain free^a^
Normal life^b^	Capability^b^	Therapy burden	Capability^b^	Drug effectiveness^b^
Support^b^	Good doctors^b^	Counselling^b^	Enjoy life^b^	Family^b^
Enjoy Life^b^	Good health^b^	Enjoy life^b^	Support^b^	Normal life^b^
Access to medicines^c^	Normal life^b^	Good care^c^	Good care	Access to medicines^c^
Fear^c^	Pain free^b^	Good doctors^c^	Good health^c^	Cure^c^
Good doctors^c^	Relapse^b^	Not to worry^c^	Good information^c^	Finances^c^
Capability^d^	Worry^b^	Pain free^c^	Access to medicines^d^	Good care^c^
Friends^d^			Friends^d^	Good health^c^
Good health^d^			Pain free^d^	Uncertainty^c^
No anxiety^d^				
Patient network^d^				
Positive mood^d^				
Work^d^				

^a^The same number of respondents reported this aspect to be important in their HRQoL; ^b^The same number of respondents reported this aspect to be important in their HRQoL; ^c^The same number of respondents reported this aspect to be important in their HRQoL; ^d^The same number of respondents reported this aspect to be important in their HRQoL

As part of the survey, respondents were asked to rate the relevance of the questions originating from the EORTC QLQ-C30 to their or the patients’ HRQoL (see Additional file 1: Appendix 2). It can be seen that in our study sample patients with a different disease stage rated different questions as relevant to their HRQoL, and that carers also seemed to rate the relevance of questions differently than patients. For example, the question in the EORTC QLQ-C30 regarding ‘Trouble doing strenuous activities’ did not seem to be relevant (at all) or did not apply to the majority of patients with stage I and II melanoma, while approximately 50% of stage III and IV melanoma patients found this a relevant question. Another example showed that the question ‘Have you had pain?’ was rated as not relevant or does not apply to the majority of stage II melanoma patients, while more than 50% of stage III melanoma patients rated this question as (very) relevant. Also 60% of carers rated this question as (very) relevant.

Table 5 provides a few examples to illustrate the extent to which current melanoma-specific HRQoL questionnaires (the EORTC QLQ-MEL38 and FACT-M) correlate to what patients indicate to be of influence to their HRQoL. Based on the aspects identified in the content analysis we determined whether a question in current melanoma-specific HRQoL questionnaires was relevant to our patient population. Additionally we assessed whether wording used in these questionnaires was similar to how patients describe this aspect in the survey. Some questions in the EORTC QLQ-MEL38 and FACT-M were relevant to our study population, while other questions seemed less relevant. For example, one question in the EORTC QLQ-MEL38 focused on patients being given enough time to think about the treatment options available. However, patients seemed to be more interested in discussing access to adequate and clear information on treatment options. Additionally, wording of questions posed in HRQoL questionnaires may differ from how patients interpret these questions. For example, questions regarding pain at the melanoma site, surgical site or headaches posed in the HRQoL questionnaires seemed to be aspects of pain that our study population did not focus on. Instead, respondents discussed pain in more general terms (e.g. future pains or experiencing pain). Additionally, while 14 of the 89 respondents discussed pain, 33 respondents focused more on being pain free as important for their HRQoL.

**Table 5:** Examples to illustrate the extent to which questions from melanoma-specific HRQoL questionnaires correlate to aspects identified by patients and carers as important to their HRQoL, based on content analysis of survey responses

Questionnaire	Question	Relevance to patient population	Difference in wording	Example of patient response
EORTC QLQ-MEL38	Have you felt able to carry on with things as normal?	Relevant	Wording similar	‘Wish to continue life as before.’
				‘Ability to life my life as normal as possible.’
EORTC QLQ-MEL38	Have you felt confident that a psychological support service would be available if you needed it?	Relevant	Wording similar	‘Care and mental support (professionals and personal network).’
				‘Piece of mind that help is just at the end of a phone.’
EORTC QLQ-MEL38	Have you received realistic and reliable information about the extent (spread) of your disease?	Relevant	Wording may differ	‘More facts and less fantasy. I could need statistics and knowledge.’
				‘Not being treated like a passive idiotic patient but being informed according to my intellectual and emotional needs.’
EORTC QLQ-MEL38	Have you had problem with pain at or near your melanoma site?	Relevant	Wording may differ	‘Worry and fears about future pain and mortality.’
				‘Being able to live without pains.’
EORTC QLQ-MEL38	Have you been given enough time to think about the treatment options available to you?	Less relevant	Wording may differ	‘Having treatment options explained and discussed with me.’
				‘Up to date knowledge of available treatments.’
EORTC QLQ-MEL38	Have you had swelling near your melanoma site?	Less relevant	NA	NA
EORTC QLQ-MEL38	Have you felt able to accept that melanoma is a serious condition?	Less relevant	Wording may differ	‘Understanding how hard it is to live with cancer (friends, relatives and work).’
				‘Doctors who don’t take your worries seriously.’
FACT-M	I get emotional support from my family	Relevant	Wording similar	‘Being surrounded by people who support you through every step of the treatment.’
				‘Family and friends support.’
FACT-M	I worry that my condition will get worse	Relevant	Wording similar	‘Worry every time it I have to go for my liver scan.‘
				‘To be free from the constant worry and stress about mets.’
FACT-M	I have a lack of energy	Relevant	Wording may differ	‘Have the energy to play with my children not be impatient because of fatigue.’
				‘Being able to exercise fully.’
FACT-M	I am bothered by side effects of treatment	Relevant	Wording may differ	‘I’m very anxious about potential side-effects from treatment.’
				‘Being able to control drug side-effects.’
FACT-M	I have good range of movement in my arm or leg	Less relevant	NA	NA

NA, Not Applicable (e.g. respondents did not discuss anything regarding this question)

## Discussion

In this study, the feasibility of using social media as a means to collect patient and carer perspectives on HRQoL was explored. Within the 30 days during which the survey was posted 89 full responses were received, showing the potential of using social media as a recruitment method. The majority of respondents accessed the survey via Facebook. Respondents resembled the general melanoma population in some aspects (e.g. melanoma stage distribution, overall HRQoL) but not others (e.g. gender distribution, educational level, geographic spread). Patients with different stages of melanoma and carers rated the relevance of questions posed in the EORTC QLQ-C30 differently. Qualitative analysis showed that some questions from the melanoma-specific EORTC QLQ-MEL38 and FACT-M questionnaires were relevant and others less relevant to our study population. Also, wording used in these questionnaires were sometimes different from how patients discussed these aspects.

Social media has been shown to provide a cost-saving and time-efficient manner to assemble valuable data [22, 36, 37]. Additionally, responses from audiences not usually included in randomised controlled clinical trials (RCTs) (e.g. women or patients with early stages of melanoma) can be collected [38, 39]. The geographic spread of patients reached through social media is considerable, ranging in this study from the United States of America (U.S.A.), to Norway, Serbia and Romania. Moreover, data collection through social media allows patients the option to provide information at their own pace and within a trusted environment of their own choice. On the other hand, not all patients will have access to the internet [36, 40], the population of patients using the internet may not reflect all patients [16, 18, 20–22], information bias may occur (e.g. duplication of social media messages or multiple messages from the same patient) [16–19], interpreting messages posted by patients may prove to be difficult for researchers, and it may be difficult to validate the authenticity of respondents via social media [16]. Keeping these disadvantages in mind, it should be emphasized that data collected through social media should be used complementary to traditional methods or provide insights where no data is otherwise available. The advantages of social media use may help increase the impact of HRQoL on REA of drugs by: increasing availability of HRQoL data for HTA, widening the scope of information from a broader patient group and increasing candidness of responses collected.

Findings from this study illustrated a difference between what patients and carers may regard as important aspects for HRQoL. Similar findings have been reported in previous research exploring responses of patients and carers to validated HRQoL questionnaires [41]. Despite the efforts invested by stakeholders to develop HRQoL questionnaires, it can thus be argued that they may not be equally implementable across patients and carers. Moreover, differences on important aspects of HRQoL extended to patients’ disease stage. Comparable findings in previous research have enticed discussions for the development of individualised HRQoL questionnaires [13, 42, 43]. This raises the question of which form of HRQoL questionnaires HTA agencies should resort to within REA’s. Moreover, it raises doubts as to whether current questionnaires are sufficient to distinguish between HRQoL of patients with different stages of melanoma. In fact, the incremental value of cancer- or melanoma-specific questionnaires for REA’s may be questionable when compared to more general tools such as the EQ-5D, considering the fact that even they may be unable to distinguish between the HRQoL of patients with different disease stages. Provided that innovative, expensive drugs are targeted at stage III/IV patients (i.e. metastatic melanoma), as well as the marginal relative incremental gains in overall survival amongst innovative drugs and toxicity profiles associated with their use, it may therefore become necessary to develop separate stage-specific HRQoL questionnaires for patients and carers to better delineate HRQoL gains with new treatments in the future [44, 45].

Meanwhile, findings on the varying relevance of questions posed in available cancer-specific or melanoma-specific questionnaires to patients may provide insights as to why completion rates for HRQoL questionnaires remain low, whether in the setting of RCTs or routine practice [5, 46]. Controversy regarding the relevance of questions posed in HRQoL in comparison to patient needs has been repeatedly cited in literature on several disease areas [13, 14, 47]. A possible explanation for this phenomenon may be that HRQoL questionnaires are conventionally developed with a physician- or scientific focus whereby the emphasis is set on aspects such as reliability, validity, and cross-cultural relevance, rather than a patient-centred approach which elicits thorough patient input at all stages of development [13, 14, 48]. The subsequent irrelevance of certain questions, in combination with factors such as disease burden and practical difficulties associated with completing paper-based questionnaires, may result in patients feeling less inclined to provide responses. Consequently, a paucity of HRQoL data for purposes such as REA ensues. If developers of new HRQoL questionnaires would address abovementioned limitations of current ones, it may thus be worthwhile to use insights provided by patients and carers through social media to ensure that the newly developed questionnaires are deemed relevant to their personal perspectives, thereby encouraging them to complete such questionnaires.

### Strengths

This study has several strengths. First, three different social media channels were used to distribute the survey, representing two different forms of social media: Twitter (micro-blogs), Facebook and LinkedIn (social networking sites). Second, open-ended questions were used in the survey, allowing respondents the opportunity to express which aspects were of influence to their HRQoL in their own terms and length. This ensured that responses compiled were likely to represent the views of their writers accurately and comprehensively. Third, two researchers conducted inductive content analysis, independently, on free text to assess the survey responses. This approach avoids limitations associated with computerised approaches such as missing misspelled words or misinterpreting slang and sarcasm. Moreover, all discrepancies related to the analysis were resolved by consensus amongst both researchers to ensure validity. Fourth, responses by patients were stratified by disease stage to highlight any potential differences in what patients may deem relevant to HRQoL per stage. Due to the low number of survey respondents, results are merely indicative of differences and inform hypotheses generation for future research.

### Limitations

A few limitations can be identified in this study. First, the survey was developed and written in English. This was not the native language of a considerable number of the respondents in this study, which may have impacted their ability to adequately represent their thoughts on the issues raised. Additionally, this may have led to selection bias since 50% of respondents were English-speaking. Second, this study provided a cross-sectional analysis of melanoma patient perspectives on HRQoL. Although this information is valuable in the context of this feasibility study, HTA decision-making on the effectiveness of melanoma drugs in practice conventionally requires longitudinal data collection on HRQoL. Therefore, the current study does not shed light on potential attrition rates in questionnaire completion or the robustness of findings from longitudinal data collection through social media. Third, the comparison of patient and carers´ perspectives on HRQoL was performed against three validated cancer- and melanoma-specific questionnaires. Other generic HRQoL instruments exist which were not included, such as the SF-36 and EQ-5D questionnaires. Provided the relevance of such generic measures for REA of drugs, this may impact the relevance of findings for HTA. On the other hand, it may be argued that the relevance of such generic measures for the comparison made would have been predictably lower than for the selected disease-specific instruments.

## Conclusion

Social media may provide a valuable tool to assess patient and carer perspectives on HRQoL, thus potentially increasing the availability and impact of HRQoL data in REA of drugs. However insights gleaned through social media are not easily generalizable to the broader melanoma patient population. Differences emerge between what patients of varying melanoma stages and carers consider important for HRQoL. Cancer- and melanoma- specific HRQoL questionnaires currently available do not seem to correlate fully with what patients view as important in HRQoL, particularly in relation to wording of issues. This raises the question of how information generated from current cancer- and melanoma-specific HRQoL questionnaires could be used for HTA decision-making and whether new, patient-centred, stage-specific instruments should be developed that better reflect patient perspectives on HRQoL.

Furthermore, current knowledge on the potential approaches for using social media to inform HTA decision-making is sparse. Although this study sheds light on the potential use of social media as a medium for gathering cross-sectional data on melanoma patient perspectives on HRQoL through questionnaires, future research should also aim to address the wide array of other potential uses, such as: the use of social media to collect longitudinal data on HRQoL, the use of data-mining approaches to glean insights on HRQoL from other channels (e.g. patient forums) and the methods for combining the potential value of the two different approaches for the use of social media (i.e. as a medium vs. data mining) for HTA decision-making. Additionally, since this was a feasibility study, a similar study on larger scale would allow for robust quantitative analysis of aspects that are important to the HRQoL of melanoma patients.

## Additional file

Additional file 1: Appendix 1. The Melanoma Quality of Life Survey: a 25-item web-based survey. Appendix 2. Relevance of questions from EORTC QLQ-C30 questionnaire in our study population, for each question in the EORTC QLQ-C30 questionnaire the percentages are calculated per stage. (DOCX 52 kb)

## Abbreviations

EORTC-MEL-38: European Organization for Research and Treatment of Cancer Quality of Life Module for Melanoma
EORTC-QLQ-C30: European Organization for Research and Treatment of Cancer Quality of Life Core Questionnaire
EQ-5D: EuroQoL 5 Dimensions
FACT-M: Functional Assessment of Cancer Therapy – Melanoma
HRQoL: Health-Related Quality of Life
HTA: Health Technology Assessment
IMI: Innovative Medicines Initiative
IMI-HARMONY: Innovative Medicines Initiative - Healthcare Alliance for Resourceful Medicines Offensive Against Neoplasms in Hematology
IMI-ROADMAP: Innovative Medicines Initiative - Real-World Outcomes Across the AD Spectrum for Better Care
MPNE: Melanoma Patient Network Europe
REA: Relative Effectiveness Assessment
