# Supplementary material for: Social media as a tool for assessing patient perspectives on quality of life in metastatic melanoma: a feasibility study
Source: Health Qual Life Outcomes. 2018 Nov 29;16:222. doi: 10.1186/s12955-018-1047-z (PMC6267816; doi:10.1186/s12955-018-1047-z)
Supplement: Supplementary file 1 — Appendix 1. The Melanoma Quality of Life Survey: a 25-item web-based survey. Appendix 2. Relevance of questions from EORTC QLQ-C30 questionnaire in our study population, for each question in the EORTC QLQ-C30 questionnaire the percentages are calculated per stage. (DOCX 52 kb) [file 12955_2018_1047_MOESM1_ESM.docx]

Additional file 1: Appendix 1. The Melanoma Quality of Life Survey: a 25-item web-based survey

Dear Melanoma patient or carer,

What is Quality of Life in Melanoma for YOU?

This study is part of the GetReal project and is conducted as collaboration between MPNE, the Melanoma Patient Network Europe, and ZIN, the Dutch National Healthcare Institute. So far, few studies have looked at what Melanoma patients themselves consider important for their own Quality of Life. The aim of this study is therefore to find out what truly matters to the Melanoma patients reached through our network. Quality of Life data is also increasingly used for the approval and reimbursement of new therapies – so please take the time to share your thoughts! We would like to understand the influence of the Melanoma stage, the time of diagnosis, the country you live in and Melanoma therapies on the Quality of Life of Melanoma patients. We also want to see if social media could be used to collect such information on patient perspectives. More information about this collaboration can be found on our website.

This survey should take 20 minutes to complete. Your answers are confidential and we will only publish anonymous results. Insights and reports will obviously be shared via the Melanoma Patient Network Europe channels!

Thank you for you time and effort.

MPNE and ZIN

Melanoma Patient Network Europe and National Healthcare Institute

We value your opinion

| 1. Quality of Life in Melanoma – which aspects come to your mind? |
| --- |
| 1. [Open Field]  2. [Open Field]  3. [Open Field]  4. [Open Field]  5. [Open Field]  6. [Open Field]  7. [Open Field]  8. [Open Field]  9. [Open Field]  10. [Open Field] |

| 2. What is Quality of Life in Melanoma for you? |
| --- |
| [Open Field] |

| 3. On a scale from 1 to 7, please rate your/the patient’s Quality of Life today. | | | | | | | |
| --- | --- | --- | --- | --- | --- | --- | --- |
|  | 1 – poor | 2 | 3 | 4 | 5 | 6 | 7 – Excellent |
| Quality of Life | O | O | O | O | O | O | O |

| 4. The 3 things that today make your/ the Melanoma patient’s Quality of Life good |
| --- |
| 1. [Open Field]  2. [Open Field]  3. [Open Field] |

| 5. The 3 things that today make your/ the Melanoma patient’s Quality of Life good |
| --- |
| 1. [Open Field]  2. [Open Field]  3. [Open Field] |

| 6. The single thing that would improve your/ the Melanoma patient’s Quality of Life right now? |
| --- |
| [Open Field] |

We value your opinion – 2

| 7. How important are for you | | | | | |
| --- | --- | --- | --- | --- | --- |
|  | Not important at all | Not important | Neutral | Important | Very important |
| Physical well-being (e.g. energy level, nauseau, pain) | O | O | O | O | O |
| Social/ Family well-being (e.g. support from family and friends, sex life) | O | O | O | O | O |
| Emotional well-being (e.g. feeling sad or nervous, worries related to Melanoma or treatments) | O | O | O | O | O |
| Functional well-being (e.g. ability to work, sleep and enjoy life) | O | O | O | O | O |
| Other (please specify below) | O | O | O | O | O |
| Other (please specify) | [Open Field] |  |  |  |  |

| 8. Please comment on question 7 |
| --- |
| [Open Field] |

| 9. How relevant are the following aspects for you | | | | | | |
| --- | --- | --- | --- | --- | --- | --- |
|  | Not relevant at all | Not relevant | Neutral | Relevant | Very relevant | Does not apply to me |
| Trouble doing strenuous activities, like carrying a heavy shopping bag or a suitcase | O | O | O | O | O | O |
| Trouble taking a long walk | O | O | O | O | O | O |
| Trouble taking a short walk outside of the house | O | O | O | O | O | O |
| Need to stay in bed or a chair during the day | O | O | O | O | O | O |
| Need help with eating, dressing, washing yourself or using the toilet | O | O | O | O | O | O |
| Limitations in doing either your work or other daily activities | O | O | O | O | O | O |
| Short of breath | O | O | O | O | O | O |
| Pain | O | O | O | O | O | O |
| Needed more time to rest | O | O | O | O | O | O |
| Trouble sleeping | O | O | O | O | O | O |
| Feeling weak | O | O | O | O | O | O |
| Lack of appetite | O | O | O | O | O | O |
| Nausea/ Feeling sick | O | O | O | O | O | O |
| Have you vomited? | O | O | O | O | O | O |
| Were you constipated? | O | O | O | O | O | O |
| Diarrhoea | O | O | O | O | O | O |
| Tiredness | O | O | O | O | O | O |
| Did pain interfere with your daily activities? | O | O | O | O | O | O |
| Difficulty in concentrating on things, like reading a newspaper or watching television | O | O | O | O | O | O |
| Feeling tense | O | O | O | O | O | O |
| Worrying | O | O | O | O | O | O |
| Feeling irritable | O | O | O | O | O | O |
| Feeling depressed | O | O | O | O | O | O |
| Difficulty remembering things | O | O | O | O | O | O |
| Physical condition or medical treatment interfered with your family life | O | O | O | O | O | O |
| Physical condition or medical treatment interfered with your social activities | O | O | O | O | O | O |
| Physcial condition or medical treatment caused you financial difficulties | O | O | O | O | O | O |
| Other (please specify) | [Open Field] |  |  |  |  |  |

| 10. Please comment on question 8 |
| --- |
| [Open Field] |

Tell us about yourself

| 11. I am | |
| --- | --- |
| O | Female |
| O | Male |

| 12. What is your Country of Residence? |
| --- |
|  |
|  |

| 13. What is your age? |
| --- |
|  |

| 14. What is the highest level of education you have completed? |
| --- |
|  |

| 15. Where did you find this survey? |
| --- |
|  |

| 16. Your relationship to Melanoma | | | | | |
| --- | --- | --- | --- | --- | --- |
|  | Stage I | Stage II | Stage III | Stage IV | N/A |
| I am a Melanoma patient | O | O | O | O | O |
| I am the carer or a Melanoma patient whose disease is in | O | O | O | O | O |
| Other (please specify) | [Open Field] | | | | |

| 17. The Melanoma diagnosis was |
| --- |
|  |

| 18. What type of Melanoma do you or the patient have? | |
| --- | --- |
|  | |
| Other (please specify) | [Open Field] |

| 19. Which mutations does your/ the patient’s Melanoma have? | |
| --- | --- |
| O | BRAF mutant |
| O | BRAF wild-type |
| O | NRAS mutant |
| O | c-kit mutant |
| O | GNAQ/ GNA11 |
| O | I don’t know |
| O | Other (please specify) |
|  | [Open Field] |

Melanoma therapies and treatments

| 20. Did you have surgery for your Melanoma? | |
| --- | --- |
| O | No |
| O | Yes |
| If yes, what type of surgery? | |
| [Open Field] | |

| 21. Did/ do you have radiotherapy for your Melanoma? | |
| --- | --- |
| O | No |
| O | Yes |
| If yes, what type of radiotherapy? | |
| [Open Field] | |

| 22. Did/ do you have chemotherapy for your Melanoma? | |
| --- | --- |
| No |  |
| Yes |  |
| If yes, what type of chemotherapy? | |
| [Open Field] | |

| 23. Did/ do you have immune therapies for your Melanoma? (please tick all that apply) | |
| --- | --- |
| O | No |
| O | Ipilimumab/ YERVOY® - BMS |
| O | Pembrolizumab/ KEYTRUDA® - MSD |
| O | Nivolumab/ OPDIVO® - BMS |
| O | T-Veck/ Talimogene Laherparepvec/ IMLYGIC® - Amgen |
| O | Pidilizumab (CT011 anti-PD1) – Curetech |
| O | Atezolizumab (anti-PD-L1) – BMS |
| O | BMS936559 (anti-PDL1) – BMS |
| O | Dendritic Cell Vaccine – academic |
| O | Adaptive Cell Therapies like TILs (T-infiltrating Lymphocytes) – academic |
| O | Other (please specify) |
| [Open Field] | |

| 24. Did/ do you have targeted therapies for your Melanoma? (please tick all that apply) | |
| --- | --- |
| O | No |
| O | Vemurafenib/ ZELBORAF® - Roche |
| O | Dabrafenib/ TAFINLAR® - Ex-GSK, now Novartis |
| O | Trametinib/ MEKINIST® - Ex-GSK, now Novartis |
| O | Cobimetinib/ COTELLIC® - Roche |
| O | Encorafenib/ LGX8181 – Ex-Novartis, now Array |
| O | Binimetinib MEK 162 – Ex-Novartis, now Array |
| O | Other (please specify) |
| [Open Field] | |

Thank you

| 25. Anything else you would like to let us know? |
| --- |
| [Open Field] |

Thank you for helping us understand what Quality of Life means to Melanoma patients.

The results of this survey will be shared in any anonymous form with the MPNE network and the general public. To make sure you don’t miss updates, please sign up to the MPNE newsletter.

MPNE and ZIN

Additional file 1: Appendix 2. Relevance of questions from EORTC QLQ-C30 questionnaire in our study population, for each question in the EORTC QLQ-C30 questionnaire the percentages are calculated per stage

| **Question in EORTC QLQ-C30:** | **Relevance** | | | | | | |
| --- | --- | --- | --- | --- | --- | --- | --- |
|  |  | **Not relevant at all** | **Not relevant** | **Neutral** | **Relevant** | **Very relevant** | **Does not apply to me** |
| Trouble doing strenuous activities | Stage I (n=17)  Stage II (n=10)  Stage III (n=16)  Stage IV (n=28)  Carers (n=19) | 18%  40%  19%  18%  11% | 24%  10%  12%  7%  5% | 6%  20%  -  18%  21% | 12%  -  25%  29%  11% | 12%  -  31%  18%  32% | 29%  30%  12%  11%  21% |
| Trouble taking a long walk | Stage I (n=17)  Stage II (n=10)  Stage III (n=16)  Stage IV (n=28)  Carers (n=19) | 12%  40%  19%  18%  5% | 24%  30%  6%  7%  5% | 6%  -  12%  18%  21% | 6%  -  6%  29%  21% | 24%  -  38%  18%  21% | 29%  30%  19%  11%  26% |
| Trouble taking a short walk outside the house | Stage I (n=18)  Stage II (n=10)  Stage III (n=16)  Stage IV (n=28)  Carers (n=19) | 28%  40%  25%  18%  17% | 22%  20%  19%  25%  17% | -  -  6%  14%  - | -  -  19%  14%  17% | 17%  10%  12%  7%  22% | 33%  30%  19%  21%  28% |
| Need to stay in bed or a chair during the day | Stage I (n=17)  Stage II (n=10)  Stage III (n=16)  Stage IV (n=28)  Carers (n=19) | 18%  50%  25%  25%  11% | 24%  10%  12%  21%  21% | 12%  -  6%  11%  11% | 6%  10%  31%  14%  11% | -  -  6%  11%  21% | 41%  30%  19%  18%  26% |
| Need help with eating, dressing, washing yourself or using the toilet | Stage I (n=17)  Stage II (n=10)  Stage III (n=16)  Stage IV (n=28)  Carers (n=19) | 18%  70%  44%  50%  26% | 18%  -  6%  7%  16% | 12%  -  -  4%  - | -  -  25%  4%  11% | 6%  -  -  7%  21% | 47%  30%  25%  29%  26% |
| Limitations in doing either your work or other daily activities | Stage I (n=17)  Stage II (n=10)  Stage III (n=16)  Stage IV (n=28)  Carers (n=19) | 18%  60%  19%  18%  11% | 18%  -  6%  7%  - | 6%  -  12%  7%  26% | 18%  10%  31%  25%  21% | 18%  -  25%  32%  26% | 24%  30%  6%  11%  16% |
| Limitations in pursuing your hobbies or other leisure time activities | Stage I (n=17)  Stage II (n=10)  Stage III (n=15)  Stage IV (n=27)  Carers (n=19) | 6%  50%  13%  15%  5% | 12%  10%  7%  7%  5% | 12%  10%  -  7%  11% | 18%  10%  53%  33%  32% | 35%  -  7%  26%  26% | 18%  20%  20%  11%  21% |
| Short of breath | Stage I (n=17)  Stage II (n=10)  Stage III (n=16)  Stage IV (n=28)  Carers (n=19) | 18%  40%  31%  43%  22% | 29%  10%  6%  4%  11% | -  10%  12%  -  11% | 6%  -  12%  29%  - | 12%  -  19%  7%  22% | 35%  40%  19%  18%  33% |
| Pain | Stage I (n=16)  Stage II (n=10)  Stage III (n=16)  Stage IV (n=28)  Carers (n=19) | 12%  50%  25%  36%  17% | 12%  10%  6%  4%  - | 12%  -  6%  11%  11% | 19%  10%  38%  21%  11% | 6%  -  19%  11%  50% | 38%  30%  6%  18%  11% |
| Needed more time to rest | Stage I (n=16)  Stage II (n=10)  Stage III (n=15)  Stage IV (n=28)  Carers (n=19) | 6%  20%  7%  21%  16% | 19%  10%  7%  4%  16% | 12%  10%  7%  7%  5% | 12%  20%  53%  29%  26% | 25%  10%  20%  32%  21% | 25%  30%  7%  7%  16% |
| Trouble sleeping | Stage I (n=17)  Stage II (n=10)  Stage III (n=16)  Stage IV (n=28)  Carers (n=19) | 6%  20%  12%  21%  - | 6%  10%  12%  -  5% | 24%  -  25%  7%  11% | 18%  30%  38%  25%  32% | 18%  10%  12%  36%  32% | 29%  30%  -  11%  21% |
| Feeling weak | Stage I (n=16)  Stage II (n=10)  Stage III (n=16)  Stage IV (n=27)  Carers (n=19) | 6%  40%  12%  37%  11% | 19%  10%  6%  7%  5% | 25%  10%  19%  7%  5% | 19%  10%  44%  15%  26% | -  -  12%  22%  37% | 31%  30%  6%  11%  16% |
| Lack of appetite | Stage I (n=17)  Stage II (n=10)  Stage III (n=16)  Stage IV (n=27)  Carers (n=19) | 18%  50%  25%  33%  26% | 29%  -  19%  7%  5% | 18%  20%  6%  15%  11% | -  -  19%  19%  11% | -  -  12%  4%  21% | 35%  30%  19%  22%  26% |
| Nausea/ Feeling sick | Stage I (n=17)  Stage II (n=10)  Stage III (n=16)  Stage IV (n=28)  Carers (n=19) | 24%  60%  25%  32%  21% | 18%  -  19%  11%  26% | 6%  -  6%  7%  5% | 6%  10%  6%  11%  11% | -  -  25%  11%  16% | 47%  30%  19%  29%  21% |
| Have you vomited? | Stage I (n=17)  Stage II (n=10)  Stage III (n=16)  Stage IV (n=28)  Carers (n=19) | 29%  70%  50%  43%  26% | 18%  -  -  7%  21% | -  -  -  11%  11% | -  -  19%  7%  5% | -  -  6%  7%  - | 53%  30%  25%  25%  37% |
| Were you constipated? | Stage I (n=16)  Stage II (n=10)  Stage III (n=16)  Stage IV (n=28)  Carers (n=19) | 19%  60%  38%  29%  11% | 19%  10%  6%  4%  5% | 6%  -  6%  11%  26% | 6%  -  25%  18%  16% | -  -  6%  11%  - | 50%  30%  19%  29%  42% |
| Diarrhea | Stage I (n=16)  Stage II (n=10)  Stage III (n=16)  Stage IV (n=28)  Carers (n=19) | 19%  70%  38%  32%  16% | 19%  -  6%  11%  26% | 12%  -  6%  11%  5% | -  -  25%  18%  21% | -  -  6%  14%  5% | 50%  30%  19%  14%  26% |
| Tiredness | Stage I (n=16)  Stage II (n=10)  Stage III (n=16)  Stage IV (n=28)  Carers (n=19) | 12%  20%  12%  18%  11% | 6%  -  -  -  - | -  -  12%  7%  - | 50%  50%  38%  21%  32% | 12%  10%  25%  54%  42% | 19%  20%  12%  -  16% |
| Did pain interfere with your daily activities? | Stage I (n=17)  Stage II (n=10)  Stage III (n=16)  Stage IV (n=28)  Carers (n=19) | 18%  30%  25%  36%  11% | 12%  20%  6%  7%  11% | -  -  12%  11%  11% | 29%  20%  25%  7%  16% | 6%  -  12%  21%  21% | 35%  30%  19%  18%  32% |
| Difficulty in concentrating on things | Stage I (n=17)  Stage II (n=9)  Stage III (n=15)  Stage IV (n=28)  Carers (n=19) | 18%  22%  33%  11%  5% | 6%  -  -  7%  26% | 29%  11%  13%  18%  5% | 18%  22%  33%  11%  26% | 12%  22%  20%  32%  11% | 29%  22%  13%  7%  26% |
| Feeling tense | Stage I (n=17)  Stage II (n=10)  Stage III (n=16)  Stage IV (n=28)  Carers (n=19) | 6%  10%  12%  11%  - | 6%  10%  -  4%  11% | 12%  -  25%  18%  5% | 35%  30%  25%  29%  26% | 29%  30%  25%  36%  47% | 12%  20%  12%  4%  11% |
| Worrying | Stage I (n=17)  Stage II (n=10)  Stage III (n=16)  Stage IV (n=28)  Carers (n=19) | -  -  -  7%  5% | -  10%  6%  11%  16% | 12%  -  -  11%  - | 35%  30%  31%  32%  16% | 41%  40%  44%  32%  47% | 12%  20%  19%  7%  16% |
| Feeling irritable | Stage I (n=16)  Stage II (n=10)  Stage III (n=14)  Stage IV (n=28)  Carers (n=19) | -  10%  -  11%  - | 6%  10%  21%  7%  16% | 19%  20%  29%  11%  11% | 38%  10%  29%  50%  37% | 25%  20%  14%  18%  21% | 12%  30%  7%  4%  16% |
| Feeling depressed | Stage I (n=17)  Stage II (n=10)  Stage III (n=16)  Stage IV (n=28)  Carers (n=19) | -  10%  -  4%  5% | 6%  -  12%  18%  16% | 12%  20%  25%  21%  5% | 35%  20%  19%  18%  12% | 18%  30%  38%  21%  42% | 29%  20%  6%  18%  11% |
| Difficulty remembering things | Stage I (n=15)  Stage II (n=10)  Stage III (n=16)  Stage IV (n=27)  Carers (n=19) | 7%  10%  19%  19%  11% | -  20%  19%  7%  21% | 27%  -  19%  19%  11% | 33%  10%  19%  19%  21% | 7%  30%  12%  22%  16% | 27%  30%  12%  15%  21% |
| Physical condition or medical treatment interfered with your family life | Stage I (n=16)  Stage II (n=10)  Stage III (n=16)  Stage IV (n=28)  Carers (n=19) | 6%  20%  12%  7%  5% | 6%  -  -  -  5% | 6%  20%  25%  21%  5% | 19%  20%  25%  32%  32% | 25%  20%  25%  32%  32% | 38%  20%  12%  7%  21% |
| Physical condition or medical treatment interfered with your social activities | Stage I (n=16)  Stage II (n=10)  Stage III (n=16)  Stage IV (n=28)  Carers (n=19) | 6%  20%  12%  4%  5% | 6%  -  -  -  5% | 6%  -  12%  18%  5% | 25%  30%  31%  29%  32% | 31%  20%  31%  36%  32% | 25%  30%  12%  14%  21% |
| Physical condition or medical treatment caused you financial difficulties | Stage I (n=18)  Stage II (n=10)  Stage III (n=16)  Stage IV (n=28)  Carers (n=19) | 12%  30%  19%  11%  11% | 12%  -  12%  7%  16% | -  10%  12%  25%  - | 24%  20%  19%  25%  11% | 18%  20%  19%  18%  42% | 35%  20%  19%  14%  21% |
